# How pollinator movement patterns emerge from the interaction between cognition and the environment

**DOI:** 10.1098/rspb.2024.2271

**Published:** 2025-04-09

**Authors:** Juliane Mailly, Louise Riotte-Lambert, Mathieu Lihoreau

**Affiliations:** ^1^ Research Center on Animal Cognition (CRCA), Center for Integrative Biology (CBI); CNRS, Toulouse University, Toulouse, France; ^2^ CEFE, CNRS, Univ Montpellier, EPHE, IRD, Montpellier, France

**Keywords:** pollination, individual-based model, traplining, behavioural ecology, bee foraging

## Abstract

Nectar-feeding insects, birds and mammals develop complex foraging patterns, such as repetitive multi-destination routes known as ‘traplines’. While this behaviour likely influences animals’ foraging success and plant mating patterns, its drivers and prevalence across species and environments remain poorly understood. Through a systematic literature review, we show that pollinators display varying degrees of movement repetitiveness. Then, using a cognitively realistic agent-based model that we parametrized with data from bee foraging studies, we demonstrate how the interplay between cognition, competition, resource distribution and nectar renewal rate can generate various foraging patterns. Our model predicts greater movement repetitiveness when floral resources are scarce and spread in space, nectar renews quickly and competition is low. These findings challenge assumptions about the prevalence of strict traplining in behavioural studies and random pollinator movements in pollination models. We discuss how a deeper understanding of the diversity of pollinator movements can improve predictions of plant mating patterns to inform precision agriculture and conservation efforts.

## 1. Introduction

Understanding how pollinators choose flowers, how frequently they visit them and in which order, is crucial to study and manage pollination. Flower visitation sequences, in particular, directly influence pollen dispersal and plant reproductive success [1]. While this level of complexity is often overlooked in current pollination models [2,3], multiple lines of experimental evidence show that pollinators do not move randomly between plants [4,5]. Instead, they develop spatial memories that guide non-random movements enabling them to revisit familiar sites, like many other animals [6]. Such movement recursions may significantly constrain pollen dispersal within the home ranges of pollinators and impact plant mating distance, mate diversity and self-pollination rates [7].

Early studies of pollinator foraging movements were interpreted in the light of optimal foraging theory, which assumes that foragers optimize their net intake rate [5,8,9]. Strikingly, many pollinators have been observed repeating sequential visits to a series of feeding sites—a behaviour called ‘traplining’ [10,11]. This routing behaviour was first considered a fixed foraging strategy and studies strived to characterize its efficiency [12–14]. However, experimental studies have shown how traplines emerge through continuous iterative changes in foraging decisions [15,16]. In this process, foragers appear to develop near-optimal routes, suggesting that traplining is the consequence of a foraging optimization mechanism based on learning [15,17,18].

Strict traplining—perfect route repetition over multiple successive foraging bouts—was mainly observed in experiments with a small number of controlled feeding sites (e.g. feeders, potted plants), a predictable renewal rate of nectar resources and little to no competition [19–21]. While this is an important first step towards a better understanding of trapline foraging, these observations do not reflect the complexity of most natural habitats and these findings may thus not be generalizable to all environmental conditions. For example, bumblebees tend to stabilize their sequences of visits to feeding sites into highly repetitive traplines within a few tens of foraging trips when foraging alone in an array of artificial feeders [15]. However, when foraging in natural habitats, they use more diverse and less repetitive foraging patterns [22], suggesting that the enhanced complexity of food resources in the field increases the variability of movement sequences.

Here, we hypothesized that pollinators, in their natural habitat, can exhibit a wide range of movement patterns—from highly variable to stable visitation sequences of feeding sites—and that these different behaviours can emerge from the interplay between cognitive abilities and environmental conditions. To test this hypothesis, we first assessed the repetitiveness of foraging routes in experimental and natural conditions with a systematic review of the scientific literature on animal movement repetition. We then explored how cognitive and environmental conditions can affect the repetitiveness of visitation sequences of feeding sites by individual pollinators using an individual-based model of bee movements.

## 2. Methods

### (a) Systematic review of the literature

In order to characterize movement repetitiveness in foraging animals, we targeted all published scientific articles that recorded foraging movement sequences of individual animals (visitation order or trajectory) and that evaluated the degree of repetitiveness of sequences of visits to feeding sites. We included both pollinator and non-pollinator species to allow for comparisons. We gathered the studies using complementary tools: Web of Science, Pubmed, Connected Papers and our pre-existing knowledge. We performed three advanced Web of Science searches: Movement* AND Rout* AND (Forag* OR Feed*) AND (Repetit* OR Repeat*); (Traplin*) and (Forag* AND Move* AND Sequence* AND (Repet* OR Repeat*)). For PubMed, we used the keywords ‘traplining’ and ‘recursion movement ecology’. For Connected Papers, we used two seminal papers from Thomson [19] and Lemke [23], Berger-Tal & Bar-David [24]’s review and Dubois’s PhD dissertation [25]. This initial phase led to a total of 539 studies.

We then excluded studies in which animals visited fewer than two feeding sites per foraging trip, as our focus was on movement sequences. We also excluded studies focusing solely on movement recursions to feeding sites without considering the order of revisits (see detailed inclusion–exclusion criteria in electronic supplementary material, table S1 and the number of studies included at each stage in electronic supplementary material, figure S1). Fifty-three articles met the inclusion criteria and were included in the review (listed in electronic supplementary material, table S2). When studies reported different experimental conditions, these conditions were considered as independent sub-studies (*n* = 86 sub-studies in total; see electronic supplementary material, figure S2 for details).

For all 86 sub-studies, we recorded the following information, if available: taxon, diet, sample size, study setting (observational or experimental), location (indoor or outdoor), number of feeding sites available in the environment, type of feeding sites (natural or artificial), renewal of feeding sites (natural, after each foraging trip, continuously, etc.), distance between neighbouring sites, measurement tools (visual observations, GPS, radar tracking, etc.), and metrics used to characterize movement sequence repetitiveness. In some cases, only an estimate of the number of feeding sites or the distance between neighbouring sites could be recorded (e.g. when a map of the environment was provided). We considered a study as ‘observational’ if the animal was observed in its natural habitat without any alteration or manipulation. A study was considered as ‘experimental’ if the animal was tested experimentally (i.e. manipulating part of the animal’s environment by setting the position of feeding sites or controlling its experience).

**Figure 1.**
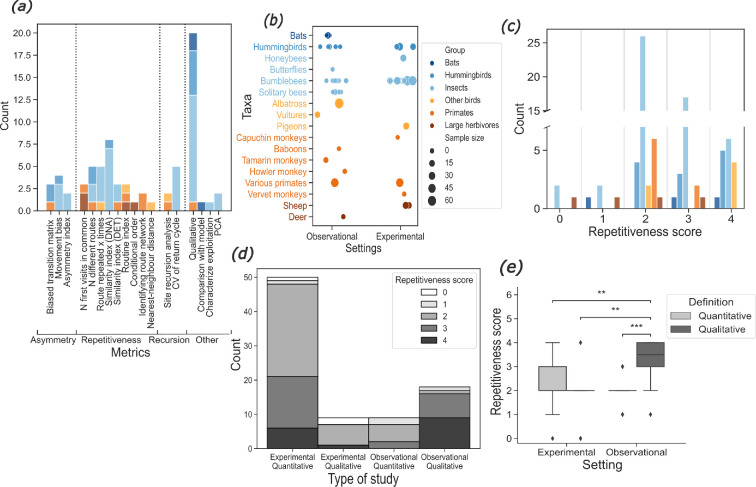
Results of the systematic review of the literature. Taxa are colour-coded by species group so that nectarivore species (bats, hummingbirds and insects) are in shades of blue, and non-nectarivore species (other birds, primates and large herbivores) are in shades of orange. (*a*) Distribution of the repetitiveness metrics used in the studies (one study can appear several times; see Methods). The metrics are grouped into four categories (asymmetry, repetitiveness, recursion and other). (*b*) Categorical scatter plot showing the different taxa against each study’s setting (natural observation versus experiment). The size of the dots represents the sample size of the study. Jitter was added to the data to avoid overplotting. (*c*) Histogram of the repetitiveness scores of all studies by species group. No significant pairwise differences were detected (Kruskal–Wallis and pairwise post hoc Dunn’s tests). (*d*) Stacked histogram and (*e*) box plot of the repetitiveness score of studies depending on the setting (observational versus experiment) and the definition of repetitiveness (qualitative versus quantitative). Pairwise significant differences are denoted by stars (Kruskal–Wallis test and pairwise post hoc Dunn’s test with Bonferroni correction; **: *p* ≤ 0.01; ***: *p* ≤ 0.001).

It was not possible to adopt a quantitative approach for two reasons. First, many studies used a qualitative assessment of traplining (see figure 1*a*), which generally implied a visual assessment of the behaviour and no data for us to analyse. Second, for the studies that reported quantitative results, many different metrics were used, and raw data were rarely provided. This prevented us from reanalysing the data with a single metric, which would have allowed for statistical comparisons between studies. We thus often had to rely on the researchers’ description of their results to classify the strength of movement repetitiveness. To compare the degree of repetitiveness of movement sequences across studies, we therefore assigned a qualitative repetitiveness score to each sub-study ranging between 0 and 4 (see table 1 for the full description criteria used to assign scores). A score of 4 corresponded to strict traplining (all the recorded feeding sites were repeatedly visited in the same order). A score of 3 denoted flexible traplining (the recorded feeding sites were repeatedly visited in the same order with some variation). A score of 2 corresponded to a significant level of routine (levels of repetitiveness were higher than expected by chance without falling into the definition of scores 3 and 4). Studies that only characterized recursions to feeding sites without reaching the criteria of score 2 were given a score of 1. A score of 0 was given to studies that did not report any form of movement repetitiveness.

**Table 1.** Grading criteria for the repetitiveness score.

Repetitiveness score	Criteria
4. Strict traplining	Strict repetition of one single route OR Clear mention of trapline without any other specification OR Route has been repeated at least three times in a row OR Similarity/routine index above 0.9
3. Flexible traplining	Does not meet criteria for a score of 4 AND Routine/similarity index above 0.7 OR Mention of entire portions of routes repeated OR Route that can be modified at times (e.g. exploration bouts) OR Reuse of a small number of routes
2. Significant routine	Does not meet criteria for a score of 3 AND Routine is higher than expected by a null model OR Results conclude in favour of movement routine or repetitiveness
1. Recursions	Does not meet criteria for a score of 2 AND Mentions revisits to feeding sites OR Quantitative characterization of recursions (i.e. revisits to feeding sites)
0. No routine	Does not meet criteria for a score of 1 AND Clear mention of no routine happening OR No difference with a null model

### (b) Individual-based model and output metrics

Our model simulates the behaviour of one or more central-place foraging pollinators exploiting simultaneously a set of feeding sites. A feeding site is a location providing large amounts of nectar on a regular basis, for example a feeder, an inflorescence, a blooming plant or a small aggregation of flowers. Here, we explored the behaviour of bees foraging for nectar by using parameter values drawn as much as possible from the bee foraging literature. The model is adapted from Dubois *et al*. [26], which uses a step-by-step decision-making process (the decisions happen at each transition between feeding sites). This model is based on recent findings that the foraging routes of bees emerge after stabilization of route ‘legs’ between two feeding sites or a feeding site and the colony nest [16,17]. These findings are consistent with the current knowledge on insect cognition, according to which path integration and visual memories underpin vector-based navigation [27,28]. We thus assume that trapline formation occurs through reinforcement learning of transition vectors linking two locations and that decisions are taken at each feeding site. This also agrees with the fact that many bees have been observed forming traplines by following local rules such as transitions between nearest neighbour feeding sites [16,17]. To adapt Dubois *et al*.’s model [26] to our needs, time is discretized thereby enabling an incremental replenishment of the feeding sites with nectar through time. We thus use a reinforcement learning algorithm that can operate on continuous rewards (Q-learning) [29]. We also add a working memory that inhibits returns to feeding sites visited a short time ago [30]. Our algorithm is fully described hereafter. A standardized description (ODD) [31] is freely available online [32]. All simulations and analyses were performed using Python v. 3.8.5 [33] and the code can be found in a public repository [34].

#### (i) Environment

The environment is a square area with continuous spatial coordinates. It contains the nest at (0;0), and a set of feeding sites. Each feeding site is characterized by its coordinates and the quantity of nectar it contains at each timestep. If the site gets emptied by a bee, the nectar is renewed linearly in a fixed number of timesteps until reaching its maximal value.

The site positions have *x* and *y* coordinates chosen from uniform distributions, except as noted. In these exceptional cases, the feeding sites are distributed in a patch [35]. To generate this patch of feeding sites, the *x* and *y* coordinates of the centre of the patch are first drawn from a uniform distribution, with a buffer around the edges of the environment to ensure that all feeding sites are generated within the environment. Then, each of the *N* sites in the patch is generated within a radius of $\sqrt{N/\pi d}$ from the centre of the patch where *d* is the density of the patch and π≃3.14. We use the average distance between feeding sites as a proxy for the density of the patch in the corresponding figures. We compute this average distance on 10 000 environments that were randomly generated with the same *d* and N parameters.

#### (ii) Behaviour

Bees move between feeding sites or between the nest and a feeding site in a straight line, with constant speed. Bees’ crop capacity is equivalent to the full nectar loads of five feeding sites. Each bee performs a series of foraging bouts, which consist in leaving the nest and successively feeding on different feeding sites until the bee’s crop capacity is full or a maximum flying distance has been reached, and returning to the nest. The bee then waits for a fixed amount of time in the nest before starting another foraging bout. We assume that the bee knows the position of each feeding site and how to move between each pair of feeding sites.

#### (iii) Learning and decision-making

Every time a bee makes a transition between feeding sites A and B, it perceives a value $v_{t}\left(A,B\right)=n_{t}\left(B\right)\times p_{d}\left(A,B\right),$ where $n_{t}\left(B\right)$ is the nectar available in site B at time *t* and $p_{d}\left(A,B\right)$ approximates the probability of discovering site B from site A following a random walk. The probability of discovering site B starting from site A following a random walk depends on the environment configuration [36]. Dubois *et al*. [26] found that using a normalization of $1/d{\left(A,B\right)}^{2}$, where *d* is the distance between feeding sites A and B, provides a close approximation of the probability while being computationally inexpensive. Thus, here $p_{d}\left(A,B\right)$is proportional to $1/d{\left(A,B\right)}^{2}$, and it is normalized so that$\sum p_{d}\left(A,\;.\right)$ equals 1 [26]. We thus assume that the initial difficulty to find feeding sites modulates the subsequent value the bee allocates to transitions.

We set the initial expected transition value between any two sites A and B as $Q_{0}\left(A,B\right)=\frac{1}{2}n_{max}\times p_{d}\left(A,B\right)$, where $n_{max}$ is the maximal nectar capacity of a feeding site. On subsequent realizations of the transition, the bee updates the expected value Q(A,B) so that $Q_{t}\left(A,B\right)=\;\alpha v_{t}\left(A,B\right)\;+\;\left(1-\alpha \right)Q_{t-1}\left(A,B\right)$ where α is the learning rate of the bee, ranging between 0 and 1. If α is smaller (respectively, larger) than 0.5, the bee relies more (respectively, less) on its previous expectation than on the newly experienced value.

Every time the bee visits a feeding site, it chooses the next feeding site to visit based on its expected values, using a softmax function as described hereafter: When on feeding site A, the probability to choose to go to feeding site B is $p_{c}\left(A,B\right)\;=exp\left(\beta Q\left(A,B\right)\right)/\sum exp\left(\beta Q\left(A,\;.\right)\right)$. β≥0 is the exploration–exploitation parameter. It controls how noisy a bee’s decision is. The closer to 0, the more the bee chooses ‘at random’ between the different options. The higher the β, the more the bee tends to choose the transition with the largest expected nectar value. β has a strong quantitative effect on the degree of routine of the simulated bee, but does not qualitatively change the effect of the other parameters of interest (electronic supplementary material, figures S3–S7). Thus, all figures are shown with an arbitrary value of β=20.

#### (iv) Memory

We assume that bees have a long-term memory (i.e. memory lasting for the whole duration of the simulation) of the positions of the feeding sites [19] and of the expected values associated with each transition between feeding sites. They also have a working (i.e. short-term) memory that inhibits the return to feeding sites visited a short time ago [30]. The span of the working memory represents the time during which recently visited sites are excluded from the transition options in the bees’ decision-making process.

#### (v) Choice of parameters

All environmental and cognitive parameter value ranges and justifications can be found in electronic supplementary material, table S3. Most parameter values are extracted from the literature. Other parameter values are chosen so that they are coherent with one another. Notably, the working memory, the time spent in the nest and the renewal duration of nectar are on a similar time scale.

#### (vi) Movement sequence repetitiveness: similarity index

We use a metric that quantifies to what degree two consecutive movement sequences a and b share smaller subsequences [26]. Consider two visitation sequences $a={Na}_{1}a_{2}...a_{p}N$ and $b=Nb_{1}b_{2}...b_{q}N$ where N is the nest, p and q the respective lengths of sequences a and b excluding nest visitations and $a_{i}$ (respectively $b_{j}$) the index of the *i*th (respectively *j*th) flower visited in sequence a (respectively b). Let us decompose sequences a and b into subsequences of size n (<p and q) with a sliding window, excluding nest visitations. We denote these subsequences $s_{a,n,k}$ for sequence a (with k ranging between 1 and p-n+1) and $s_{b,n,l}$ for sequence b (with l ranging between 1 and q-n+1) such that ${s_{a,n,1}=a}_{1}a_{2}...a_{n}$; ${s_{a,n,2}=a}_{2}a_{3}...a_{n+1}$; etc., and ${s_{b,n,1}=b}_{1}b_{2}...b_{n}$; ${s_{b,n,2}=b}_{2}b_{3}...b_{n+1}$; etc. We then count the number of unique visitations $a_{i}$ and $b_{j}$ that belong to subsequences that appear both in a and b, i.e. for which there exist an index k and an index l such that $s_{a,n,k}=s_{b,n,l}$. We denote this count $S_{ab}$. The similarity index ${SI}_{ab}$ is then defined as $SI_{ab}=S_{ab}/2l_{ab}$with $l_{ab}$ = max(*p*, *q*). The length of the subsequences n is set at 3 as in Dubois *et al*. [26], which is long enough to detect subsequences but not too large compared with the length of a single foraging trip (five full-site visits). When two consecutive foraging bouts have no subsequence in common, the similarity index equals 0. If two consecutive foraging bouts are identical, the index equals to 1.

#### (vii) Foraging success

To assess the foraging efficiency of a bee, we measure the quantity of nectar per unit of time collected during each foraging bout in µl s^−1^ [37].

#### (viii) Figures

Each data point in the figures is the average of 50 different simulations on 25 different environment configurations (arrays). To allow bees sufficient time to reach their full routine potential following the initial learning phase, simulations are run for an extended period (7 h of foraging, i.e. about one foraging day), ensuring stabilization of the average similarity index and foraging success values, as this period is much shorter than the two weeks of foraging activity allowed by the life expectancy of a worker bee [38]. Figures are displayed at a time (foraging bout) for which both metrics have reached a plateau for all the conditions represented in the figure. This time is chosen after looking at the temporal evolution of the two metrics and visually confirming that all conditions have reached a plateau. The chosen time can differ between figures. Full plots showing the metric values through time are available in electronic supplementary material, figures S8–S11.

## 3. Results

### (a) How repetitive are pollinators’ foraging sequences in field observations and experimental studies?

The literature uses a rich diversity of metrics (figure 1*a*) to describe movement repetitiveness in a variety of animal taxa. However, most studies (74%) focused on nectarivores (figure 1*b*). But repetitive movements were also reported in non-nectarivores such as monkeys [39], large herbivores [40] and birds [41] that exploit other renewing or non-depletable feeding sites like fruit trees or grass patches (figure 1*b*).

Interestingly, no animal group displayed exclusively strict traplining (repetitiveness score of 4; figure 1*c*). There was no significant pairwise difference between groups of species (Kruskal–Wallis test, *p* = 0.04; post hoc Dunn’s test with Bonferroni correction, *p* > 0.05). Overall, 47% of the 86 sub-studies had a score of 3 and above, indicating that flexible traplining is commonly found in the literature. However, only 19% of the sub-studies reported a score of 4 indicative of strict traplining.

Many studies have adopted a qualitative observational approach, through visual observations of animal occurrences or feeding site visitation sequences (figure 1*d,e*; qualitative observational settings [42]). These studies show significantly higher scores than quantitative studies (figure 1*e*; post hoc Dunn test), which monitored the foraging paths of individuals (through the use of radar, cameras, RFID, GPS, etc.) and quantified movement repetitiveness statistically. This difference suggests that qualitative observational studies overestimate the perfection of traplines.

### (b) What environmental factors can influence the repetitiveness of pollinators’ movement sequences?

Our analysis of the literature indicated that pollinators express diverse levels of movement repetitiveness (figure 1). This is coherent with previous observations showing that environmental parameters, such as the spatial distribution of food resources [20], the renewal duration of nectar [43] and competition [44], influence the foraging patterns of bees in specific experimental conditions [35]. Below we further explore the mechanisms behind these effects.

#### (i) Movement sequences are more repetitive when few, widely spaced feeding sites are available

**Figure 2.**
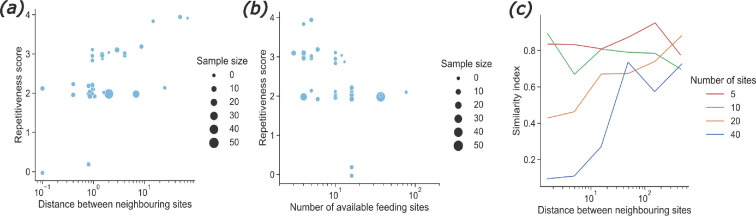
Effect of the spatial distribution of resources. Results from the systematic review. Effect of (*a*) the distance between two neighbouring feeding sites (in metres) and (*b*) the number of available feeding sites on the repetitiveness scores of the studies focusing on bees. Results from the model. (*c*) Effect of the distance between neighbouring feeding sites (*x*-axis, in metres) and the number of feeding sites available (different colours) on the similarity index, at foraging bout 10. We simulated a bee foraging alone in an environment containing 5−40 feeding sites in a patch. See electronic supplementary material, figure S2 for other values of *β*.

Pollinator movement sequences have been experimentally observed to be more repetitive when fewer feeding sites are available [21,45] and when the distances between feeding sites increase [20]. Accordingly, in our analysis of the literature, we found that the repetitiveness score of studies on bees slightly but significantly increased with the distance between neighbouring feeding sites (figure 2*a*; Kendall’s $\tau_{b}$ = 0.61, *p* < $10^{-6}$with the distance between sites in log scale) and decreased with the number of available feeding sites (figure 2*b*; Kendall’s $\tau_{b}$ = − 0.61, *p* < $10^{-5}$ with the number of sites in log scale).

Using our model, we could confirm these effects (figure 2*c*). We simulated one forager exploiting a patch of feeding sites and varied the distance between feeding sites and the number of sites in the patch. The repetitiveness of the movement sequences (similarity index—see Methods) increased with the distance between feeding sites. However, this effect was less pronounced, and even disappeared, when only a few feeding sites were available (below 10 feeding sites in figure 2*c*), indicating that the two phenomena can interact (figure 2*c*). The jumpiness of the curve in figure 2*c* is probably due to the small number of distinct environments simulated (25 per condition).

#### (ii) The repetitiveness of movement sequences decreases when nectar renewal duration increases

Resource renewal is considered one of the main drivers of routine movement behaviour [24]. However, the implications of the nectar renewal duration have rarely been studied experimentally [43]. While in most experimental studies on bees, the renewal of feeders was discrete and happened every time the forager was back in the nest [15,16,18], in natural flowers, nectar renews gradually until reaching a plateau [46]. The timing of flower revisits is thus crucial to take into account: if the pollinator returns too early, it does not maximize its nectar intake. Conversely, if the pollinator returns too late, it has no extra nectar gain and risks losing the reward to a competitor [12]. The forager can adjust this timing if it develops memories of individual visits to flowers [30].

**Figure 3.**
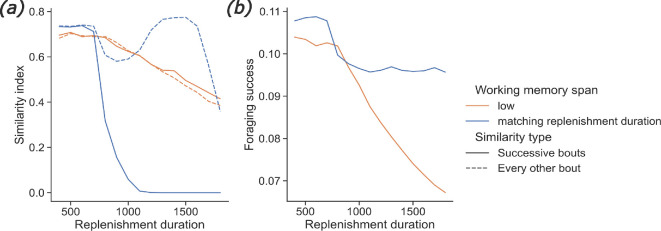
Effect of the replenishment duration. Results from the model. Effect of the replenishment duration and the span of the working memory on (*a*) the similarity index, either between two consecutive foraging bouts (bouts *N* and *N* + 1, plain lines), or between foraging bouts *N* and *N* + 2 (dashed lines) and (*b*) the foraging success, at foraging bout 15. A bee is simulated foraging alone in an environment containing 25 uniformly distributed feeding sites. The bee either has a low working memory span (30 s; orange) or a working memory span matching the nectar renewal duration of the feeding sites (blue). See electronic supplementary material, figure S3 for other values of *β*.

Simulations of our model, with continuously renewing feeding sites, predict that if the bee’s working memory is short (figure 3, orange curves), both movement sequence repetitiveness and foraging efficiency decrease with nectar renewal duration. However, if the bee’s working memory span matches the renewal duration of the feeding site, its movement patterns vary with nectar renewal durations, while the foraging success remains high (figure 3*b*). Specifically, for short nectar renewal duration values, the similarity between feeding site visitation sequences across consecutive foraging bouts is high, and therefore, the similarity between every other bout is high too. Thus, the bee uses only one route. For long renewal duration values (around 1500 s), the every-other-bout similarity is high while the similarity of consecutive bouts is low, corresponding to the bee alternating between two routes. This could result from the working memory inhibiting visitation of the flower first visited during the preceding bout when the bee starts a new foraging bout (i.e. when the working memory exceeds the duration of a foraging bout). For intermediate nectar renewal duration values, around 1000 s, the every-other-bout similarity decreases. It could be because the bee is integrating parts of a new route in its current trapline.

#### (iii) Competition decreases the repetitiveness of movement sequences

It has been suggested that traplining enables pollinators to keep flowers’ standing crops at low levels, thus acting as a ‘defence by exploitation’ strategy [12]. This hypothesis implies that movement repetitiveness should increase with the number of competitors as long as the environment is sufficiently rich to support all foragers. Only a few studies have experimentally explored the effect of competition on movement sequence repetitiveness in pollinators [44,47,48]. These experiments showed that foragers that had time to explore their environment and develop repetitive movement sequences tend to be more efficient at foraging than less experienced competitors. However, in these conditions, the presence of a competitor did not affect movement repetitiveness, nor did it promote the development of stable traplines [44,47,48].

**Figure 4.**
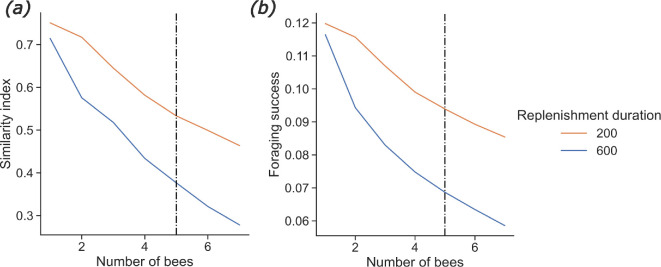
Effect of competition. Results from the model. Effect of the number of bees (*a*) on the average similarity index and (*b*) on the foraging success, at foraging bout 15. One bee colony is simulated foraging in an environment containing 25 uniformly distributed feeding sites. Altogether, these feeding sites can fill to capacity the crop of five bees (dashed vertical lines). We varied the number of foragers in the nest and the nectar renewal duration (represented by different colours). See electronic supplementary material, figure S4 for other values of *β*.

Our model provided results that do not support the defence-by-exploitation hypothesis as the increase in the number of foragers in the environment—all equally experienced and originating from the same nest—reduced route repetitiveness and foraging efficiency, even if the environment could provide food for all of them (figure 4). This effect was even more striking if the replenishment of nectar was slow (figure 4, blue curves). Such a drop in route similarity might be explained by resource depletion and a higher occurrence of competitive encounters in the vicinity of the nest. Feeding sites close to the nest are likely to be visited at the beginning of a route and any difficulty in visiting them may lead the forager to use a completely different route.

## 4. Discussion

Bees, butterflies, birds, bats and many other animals display different levels of movement repetitiveness (figure 1*c*). Our model of bee movements shows that this behavioural diversity could emerge from a simple and realistic step-by-step reinforcement learning. The various foraging patterns of pollinators can thus be seen as a continuum of movement repetitiveness—from random movements to strict traplining—that can be quantified by complementary metrics (e.g. DET [49], Routine Index [50], Similarity Index [26]). Such a conceptual representation of a continuum in movement repetitiveness could help address the challenges associated with the concept of ‘traplining’, which sparks debates about the threshold of repetition necessary to satisfy its designation [11].

Our model predicts that pollinators could produce a wide range of movement patterns depending on environmental conditions—from random movement sequences to strict traplining, and even alternating between multiple routes. High levels of movement repetitiveness are more likely to occur when feeding sites are distant from one another, when they are scarce, when the renewal of resources is fast and when there is little to no competition. This agrees with the observation that strict traplining has been reported in experimental designs where the position and renewal duration of feeding sites were controlled [15]. Such highly repetitive movements are indeed favoured in environments that do not provide many resource options to explore, thus suppressing more flexible movement patterns.

The prediction that few, distant feeding sites favour repetitive foraging patterns matches the behaviour of various pollinator species [20,21,45] and concurs with the results of our systematic review (figure 2*a*,*b*). However, to our knowledge, no study has yet investigated the effects of nectar renewal duration on large-scale foraging patterns. Bees and hummingbirds can track the time interval between flower visits [51,52] and adapt it to the renewal duration of feeding sites [53–55]. This supports the prediction that foragers might alternate between different routes and save time between repeated visits to feeding sites (figure 3). Competition is also known to influence foragers’ movement patterns [44,47] and impair the ability of individuals to forage efficiently when resource availability is limited [48]. However, these results are difficult to compare to our model’s predictions as we assumed that all foragers were equally experienced and shared the same nest (figure 4). As far as we know, there is no report of nestmate discrimination between bees in a foraging context. We thus expect that more colonies would bring more indiscriminate competitive interactions (i.e. similar to those currently implemented in the model) if the different nests are close to each other. This could be the focus of further investigation.

Our study also predicts that the diversity of foraging patterns observed in pollinators is influenced by their cognitive abilities, namely learning, decision-making and working memory. However, such complex abilities might only be useful when pollinators face specific environmental conditions. Notably, our simulations showed that a long working memory is only advantageous when the renewal of nectar is slow (figure 3). Additional simulations enabled us to compare foragers without learning capacities and with substantial randomness in their decision-making (α = 0, β = 2, see Methods) to foragers that can use both reinforcement learning and very reliable decision-making. While more advanced learning and decision-making capacities seem advantageous in environments where feeding sites are far from each other, the foraging success of the two types of foragers is similar in environments where feeding sites are very close to each other (electronic supplementary material, figures S6 and S7). These results suggest that such learning and decision-making capacities would only be advantageous for foragers that travel long distances to find feeding sites. Therefore, the cognitive abilities underlying the behavioural flexibility of our simulated pollinators might only have evolved for central-place forager species that need to forage in harsh conditions (large distance between patches, slow nectar renewal). This might be a potential explanation for the wide range of behaviours observed in pollinator species (figure 1*c*), which often have to forage in complex heterogeneous landscapes [56].

Importantly, many of these theoretical predictions can readily be tested experimentally by selectively manipulating key environmental parameters in the foraging environments of animals, such as the number of feeding sites, their spatial distribution and the density of foragers. Such investigations are made possible in semi-natural settings by recent technological advances including automated feeders [57], computer vision at feeding sites [58] and radar [59] to accurately track the flight paths of marked pollinators. Combining these complementary approaches is likely to overcome current limitations in accurately tracking individual (or a few) foragers in obstacle-free environments [15,16].

Ultimately, beyond informing about the diversity of pollinators’ foraging strategies, better characterizing their spectrum of spatial behaviours and their environmental drivers can have far-reaching applications for pollination ecology. Current pollination models rarely consider individuals’ behaviour, thereby potentially overlooking the fundamental role of learning and memory in driving pollinators’ foraging patterns and interactions [2]. Our results suggest important consequences regarding pollen dispersal and plant reproduction. For example, the predicted differences in movement sequence repetitiveness depending on the distances between feeding sites (figure 2) might trigger different pollen dispersal patterns between and within patches of flowers. We hypothesize that random movements within flower patches might cause rather diffusive, nearest neighbour pollen dispersal, whereas repetitive movements between flower patches at larger spatial scales might constrain plant gene flow within the itineraries of pollinators, provided that the foragers are spatially segregated [60]. The nectar renewal duration might also play a big part in pollen dispersal patterns. For pollinators with a short working memory, slower renewal causes less repetitive movements (figure 3), which could decrease plants’ mating distance [7]. However, slower nectar replenishment requires that pollinators visit more flowers to fill up their crops, as flowers tend to be often found empty, which could, in turn, increase mating distance [7]. These two phenomena might interact in a non-trivial way. Finally, competition might reduce movement repetitiveness (figure 4) but still cause spatial segregation [61], which implies that a given flower patch would be monopolized by a single pollinator. In that case, pollen dispersal would be constrained within the home range of a single pollinator—as opposed to many pollinators when there is no spatial segregation.

These new hypotheses, derived from model predictions of bee foraging patterns, warrant further investigation and hold considerable potential for improving pollination models. We believe mechanistic tools, such as our realistic bee movement model, could play a key role in the upcoming years amid the looming pollinator crisis. In other ecology subdisciplines, the predictions of process-based models in changing environments have been shown to be more robust than the ones derived from phenomenological approaches [62–64]. Integrating pollinator movements into pollination models offers valuable potential to improve predictions of the impacts of global change on plant reproduction and to develop effective strategies to safeguard pollinators and the critical services they provide [65,66].

## Data Availability

The repository containing the dataset and data analysis code for the literature review, as well as the model’s code and data analysis pipeline, is freely available on Figshare [34]. The full model description is also available on Figshare [32]. Supplementary figures and tables are provided in the electronic supplementary materials. Supplementary material is available online [67].
